# Hypomethylation of the promoter region drives ectopic expression of *TMEM244* in Sézary cells

**DOI:** 10.1111/jcmm.15729

**Published:** 2020-08-14

**Authors:** Katarzyna Iżykowska, Karolina Rassek, Magdalena Żurawek, Karina Nowicka, Julia Paczkowska, Iwona Ziółkowska‐Suchanek, Marta Podralska, Agnieszka Dzikiewicz‐Krawczyk, Monika Joks, Karolina Olek‐Hrab, Maciej Giefing, Grzegorz K. Przybylski

**Affiliations:** ^1^ Institute of Human Genetics Polish Academy of Sciences Poznań Poland; ^2^ Department of Hematology and Marrow Transplantation University of Medical Sciences Poznań Poland; ^3^ Department of Dermatology University of Medical Sciences Poznań Poland

**Keywords:** CRISPR‐dCas9, DNA methylation, Sézary syndrome, TET1, TMEM244

## Abstract

Sézary syndrome (SS) is an aggressive form of cutaneous T‐cell lymphoma (CTCL) characterized by the presence of circulating malignant CD4+ T cells (Sézary cells) with many complex changes in the genome, transcriptome and epigenome. Epigenetic dysregulation seems to have an important role in the development and progression of SS as it was shown that SS cells are characterized by widespread changes in DNA methylation. In this study, we show that the transmembrane protein coding gene *TMEM244* is ectopically expressed in all SS patients and SS‐derived cell lines and, to a lower extent, in mycosis fungoides and in a fraction of T‐cell lymphomas, but not in B‐cell malignancies and mononuclear cells of healthy individuals. We show that in patient samples and in the T‐cell lines *TMEM244* expression is negatively correlated with the methylation level of its promoter. Furthermore, we demonstrate that *TMEM244* expression can be activated in vitro by the CRISPR‐dCas9–induced specific demethylation of *TMEM244* promoter region. Since both, *TMEM244* expression and its promoter demethylation, are not detected in normal lymphoid cells, they can be potentially used as markers in Sézary syndrome and some other T‐cell lymphomas.

## 1 INTRODUCTION

Sézary syndrome (SS) is an aggressive, leukaemic cutaneous T‐cell lymphoma (CTCL) variant characterized clinically by severe erythroderma, pruritus and lymphadenopathy,^1^ and the presence of atypical, malignant Sézary cells in blood, lymph nodes and skin with CD3/CD4 expression and heterogeneous naïve/memory maturation phenotype.^2^ SS accounts for 4% of CTCL with the incidence of 0.4/1000 000, yet the prognosis for patients is poor (5‐year survival of 30%).^1^, ^3^ SS is characterized by many complex changes in the genome and epigenome^4^, ^5^, ^6^, ^7^ that influence the transcriptome thereby leading to malignant transformation. Epigenetic dysregulation seems to have an important role in the development and progression of SS, as it was shown that genes involved in methylation, like *DNMTs* and *TETs*, are often mutated in SS,^3^ and SS cells are characterized by widespread changes in DNA methylation,^8^ including hypermethylation of tumour suppressor genes.^9^ In cancer, disruption in methylation pattern, especially global hypomethylation of the genome, leads to chromosomal instability and consequently to altered transcription and impaired signalling pathways.^10^

In our previous study, we identified ectopic expression of transmembrane protein gene (*TMEM244*), with unknown biological function, in SS patients but not in healthy individuals.^11^ The purpose of this study was to unravel the mechanism responsible for *TMEM244* activation. We found a negative correlation between *TMEM244* expression and promoter methylation in patient samples and in T‐cell lines suggesting methylation to be a mechanism responsible for regulation of *TMEM244* expression. This concept was proved in vitro using CRISPR‐dCas9 epigenome editing system, by activating *TMEM244* expression in Jurkat cells upon specific demethylation of selected CpGs in *TMEM244* promoter region.

## 2 MATERIALS AND METHODS

### 2.1 Clinical samples

Five Sézary syndrome blood samples were included in the study: P1 (F‐female, age: 80), P2 (M‐male, age: 65), P3 (M, age: 54) from the previous study,^11^ and two new SS patients: SS1 (M, age: 85) and SS2 (M, age: 72). All SS samples were received from the Department of Dermatology, University of Medical Sciences, Poznan, Poland. Three of them were mononuclear cells (PBMCs) purified by density gradient centrifugation in Histopaque‐1077 (Sigma‐Aldrich, Germany) (P1‐P3), while two others (SS1 and SS2) were sorted CD4^+^ T cells, separated with Human CD4 + T Cell Enrichment Kit (StemCell Technologies).

Three mycosis fungoides samples (MF1‐MF3) were collected from the Department of Hematology and Marrow Transplantation, University of Medical Sciences, Poznan, Poland: MF1 (blood), MF2 (blood, bone marrow, skin biopsy) and MF3 (blood, bone marrow). CD4 + lymphocytes were separated from blood and bone marrow by density gradient centrifugation in Histopaque‐1077 (Sigma‐Aldrich) and Human CD4 + T Cell Enrichment Kit (StemCell Technologies). Lymphocytes from the skin biopsy (size: 0.5 cm^2^) were isolated as described by Salimi et al^12^

Thirteen blood samples, 3 bone marrow samples from different haematological malignancies and 1 healthy bone marrow sample (BM1) were obtained from the Department of Hematology and Marrow Transplantation, University of Medical Sciences, Poznan, Poland (Table 1). Five blood samples from healthy individuals from the previous study were used as controls (C1‐C5).^11^ PBMCs were purified from those samples using Histopaque‐1077 (Sigma‐Aldrich).

**Table 1:** *TMEM244* expression and promoter methylation in haematological patients, healthy individuals and T‐cell lines

Patient ID	Sex age	Diagnosis	WBC G/L	Lymph G/L	CD4+	CD4/CD8	Sample	Relative *TMEM244* expression 2^‐∆CT^/2^−∆∆CT^	*TMEM244* promoter region methylation (%mean ± SD)
Sézary syndrome and Mycosis fungoides									
P1	80 F	SS	24.8	18.3 (74%)	95%	15.9	PB	**371E‐6/198.84**	**33.47 ± 2.94**
P2	65 M	SS	9.5	1.5 (15%)	83%	7.2	PB	**1 150E‐6/61.50**	**67.85 ± 6.54**
P3	54 M	SS	12.5	6.1 (49%)	74%	4.5	PB	**521E‐6/27.86**	**59.86 ± 2.94**
SS1	85 M	SS	18.9	13.8 (73%)	92%	16.7	PB	**8 224E‐6/439.79**	**4.28 ± 0.70**
SS2	72 M	SS	9.3	4.5 (48%)	90%	45	PB	**173E‐6/9.25**	**33.37 ± 1.74**
MF1	76 M	MF	7.0	1.0 (14%)	52%	1.9	PB	**198E‐6/10.59**	**59.01 ± 4.2**
MF2	68 M	MF	10.2	3.0 (29%)	60%	1.7	PB	**351E‐6/10.59**	**69.99 ± 6.8**
				(13%)	55%	1.5	BM	57E‐6/3.05	83.75 ± 7.4
							SB	9E‐6/0.48	71.31 ± 13.7
MF3	39 F	MF	5.3	1.5 (29%)	54%	2.6	PB	48E‐6/2.57	75.53 ± 5.78
							BM	50E‐6/2.67	83.01 ± 1.63
Other T‐cell lymphomas and acute T‐cell lymphoblastic leukaemia									
							PB	34E‐6/1.82	84.23 ± 3.7
TCL1	47 M	EATL					BM	**234E‐6/12.51**	73.36 ± 13.9
TCL2	72 F	PTCL					PB	18E‐6/0.96	91.59 ± 4.9
TAL1	25 M	ETP‐ALL					PB	**165E‐6/8.82**	80.29 ± 23.5
TAL2	50 F	T‐ALL					PB	91E‐6/4.87	76.36 ± 4.4
TAL3	52 F	T‐ALL					LN	89E‐6/4.76	84.79 ± 17.2
							PB	34E‐6/1.82	84.23 ± 3.7
B‐cell leukaemia									
CLL1	58 F	CLL					PB	2E‐6/0.11	92.54 ± 4.7
CLL2	51 M	CLL					PB	4E‐6../.0.0.21	95.96 ± 1.5
CLL3	69 M	CLL					PB	8E‐6/0.43	83.16 ± 19.9
CLL4	73 M	CLL					PB	1E‐6../.0.0.05	92.49 ± 1.8
CLL5	73 M	CLL					PB	**433E‐6../.0.23.16**	**53.73 ± 22.5**
CLL6	61 M	CLL					PB	2E‐6/0.11	94.09 ± 2.3
CLL7	71 M	CLL					PB	24E‐6/1.28	78.74 ± 24.3
CLL8	59 M	CLL					PB	30E‐6/1.60	80.40 ± 23.3
HCL1	79 F	HCL					PB	15E‐6/0.80	93.81 ± 1.6
HCL2	43 M	HCL					BM	59E‐6/3.16	80.36 ± 7.1
BAL	22 F	B‐ALL					BM	8E‐6/0.43	91.28 ± 5.2
Healthy individuals									
BM1	63 F	HI					BM	16E‐6/0.86	91.44 ± 4.0
C1	49 M	HI					PB	23E‐6/.0.1.23	87.61 ± 5.7
C2	42 M	HI					PB	21E‐6../.0.1.12	88.83 ± 6.6
C3	50 F	HI					PB	1E‐6/0.05	84.80 ± 7.84
C4	41 M	HI					PB	15E‐6/0.80	85.72 ± 8.30
C5	42 M	HI					PB	36E‐6‐‐/1.93	86.44 ± 6.16
T‐cell lines									
SeAx		SS					CC	**2 536E‐6/135.61**	**1.9 ± 1.03**
HH		CTCL					CC	**363E‐6/19.41**	**3.2 ± 0.47**
Hut78		CTCL					CC	50E‐6/2.67	**56 ± 25.81**
HDLM2		T‐cell HL					CC	**1 482E‐6/79.25**	**1.8 ± 0.52**
Jurkat		T‐ALL					CC	0E‐6/0.00	85.5 ± 18.69

Note

Meaningful *TMEM244* expression (>100E‐6) and meaningful promoter hypomethylation (<70%) are given in bold.

Abbreviations: ALL, acute lymphoblastic leukaemia; B‐ALL, B‐cell acute lymphoblastic leukaemia; BM, bone marrow; CC, cell culture; CLL, chronic lymphocytic leukaemia; CTCL, cutaneous T‐cell lymphoma; EATL, enteropathy‐associated T‐cell lymphoma; HCL, Hairy cell leukaemia; HI, healthy individual; HL, Hodgkin lymphoma; LN, lymph node; MF, mycosis fungoides; PB, peripheral blood; SB, skin biopsy; SS, Sézary syndrome; T‐ALL, T‐cell acute lymphoblastic leukaemia.

The use of human material was approved by the Local Ethics Committee (Decision1095/17) and performed in accordance with the Declaration of Helsinki. Informed consent was obtained from all individual participants involved in the study.

Samples were used to extract DNA (Gentra Puregene Blood Kit, Qiagen) and RNA (TRI Reagent, SIGMA) according to the manufacturer's protocol. The quantity of RNA and DNA was measured using the NanoDrop 2000 spectrophotometer (Thermo Fisher Scientific™, Waltham, CA), and the quality was determined by 1% agarose gel electrophoresis with ethidium bromide staining. cDNA was synthetized from 0.5 μg of RNA using QuantiTect Reverse Transcription Kit with random hexamer primers (Qiagen, Germany).

### 2.2 Cell lines

Five established cell lines were included in the study: 3 CTCL cell lines (Hut‐78—Sézary syndrome, ATCC TIB‐161; HH—aggressive cutaneous T‐cell leukaemia/lymphoma, ATCC CRL‐2105; and SeAx—Sézary syndrome, kindly provided by Dr Markus Möbs^13^), T‐cell acute lymphoblastic leukaemia (T‐ALL) cell line Jurkat (SIGMA 88042803), and T‐cell Hodgkin lymphoma cell line HDLM2 (DSMZ ACC17). They were cultured in a HEPES‐buffered RPMI1640 medium with L‐glutamine (Thermo Fisher Scientific™), 10%‐20% foetal bovine serum (Sigma) and 1% penicillin/streptomycin (Life Technologies), according to manufacturer's instructions. Medium for SeAx was supplemented with Il‐2 (200 U/mL) (Sigma‐Aldrich) and medium for Jurkat with 1% sodium pyruvate (1 mmol\L) and 0.25% glucose (0.5 g/L) (Life Technologies).

### 2.3 Real‐time quantitative PCR (RT‐qPCR)

RT‐qPCR was performed using the CFX96 Real‐Time System (Bio‐Rad, Hercules, CA). *TMEM244* expression was analysed using TaqMan Gene Expression Assays (Applied Biosystems, Foster City, CA) (Hs02340633_m1), with intron‐spanning primers located in the second and third exons. Beta‐2 microglobulin (*B2M)* (Hs00984230_m1), with intron‐spanning primers located in the first and second exons, was used as a reference gene for sample normalization. For both genes, Applied Biosystems TaqMan MGB (minor groove binder) dual‐labelled hydrolysis probes were used, incorporating a 5' fluorescent reporter dye and a 3' nonfluorescent quencher (NFQ). All samples were assayed in triplicates, and median value was used to calculate relative gene expression, according to the Livak method (2^−∆CT^). The 2^‐∆∆CT^ Livak equitation was additionally calculated for the patient samples, using as calibrator the mean value of healthy individuals. The 2^‐∆CT^>100E‐6, corresponding to the 2^‐∆∆CT^>5.35, was considered to be significant expression of *TMEM244*.

### 2.4 Plasmids and sgRNA cloning

Two lentiviral CRISPR‐dCa9 vectors: pLV[Exp]‐Bsd‐EF1A > dCas9*:active TET1 (Tet Methylcytosine Dioxygenase 1) (ID: VB190118‐1114rnk) and pLV[Exp]‐Bsd‐EF1A > dCas9*: inactive domain TET1 (VB190118‐1116tye) were designed by VectorBuilder (Chicago, USA) (Supplementary). Both vectors have catalytically inactive Cas9 (dCas9) fused to either active or inactive (mutated) domain of TET1 demethylase.^14^

3rd generation lentiviral gRNA expression vector: pU6‐sgRNA Ef1 alpha Puro‐T2A‐GFP was a gift from dr LA Gilbert.^15^

**FIGURE 1:**
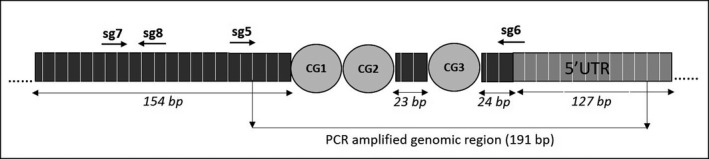
*TMEM244* promoter region. Genomic position of three CpG dinucleotides: chr6:130,182,479‐130,182,514; GRCh37/hg19; four sgRNAs are indicated by arrows

Four single guide RNAs (sg5: GAGAACTCCATCGTTTAATA; sg6: ACGCAGTAG‐TGCAGGATGAT, sg7:AATTACTCATACAGCCAGAG, sg8:GATAGTGCGGCAAATAG‐GCA) were designed using CRISPOR program (http://crispor.tefor.net/) to target the three CpG dinucleotides that were investigated for DNA methylation level. The sgRNA localization was selected in close proximity to the CpGs, to avoid binding of the large dCas9‐TET1 protein complex directly to the CpGs (Figure 1). To exclude any off‐target effects, two no‐targeting sgRNAs were used: (NT3: ACGGAGGCTAAGCGTCGCAA and NT4: ATCGTTTCCGCTTAACGGCG). For functional experiments, Jurkat cell line, not expressing *TMEM244* and showing hypermethylated promoter region of the gene, was chosen. Sense (5' TTG‐‐‐GTTTAAGAGC 3') and antisense (5' TTAGCTCTTAAAC‐‐‐CAACAAG 3') oligonucleotides were annealed and cloned into a lentiviral pU6 vector backbone using BstXi and BlpI restriction sites and respective enzymes (NEB). Annealed oligonucleotides were ligated into a vector, before the sgRNA scaffold, using T4 ligase (Promega). Plasmid was replicated in TOP10 electrocompetent E. coli (Thermo Fisher Scientific™) and purified using Qiagen Plasmid Plus Kit (Qiagen, Germany). Cloned sgRNA constructs were sequenced to confirm the correctness of the inserted sequence.

### 2.5 Lentiviral production

HEK293T (DSMZ ACC 635) cells were cultured in DMEM (Lonza) supplemented with 10% FBS (Sigma) and 1% penicillin/streptomycin (Life Technologies). Lentiviral vectors were co‐transfected with 3rd generation packaging plasmids: pMSCV‐VSV‐G, pRSV.REV, pMDL‐gPRRE into HEK293T cells using Lipofectamine 2000 (Thermo Fisher Scientific). Medium was replaced 24 hours after transfection. 48 and 72 hours post‐transfection, viral supernatant was collected, sterile filtered through 0.45 μm syringe filter and stored at −80°C.

### 2.6 Jurkat transduction

Jurkat cells were first transduced with lentivirus particles containing dCas9‐TET1 active/inactive fusion. Virus supernatant was added to cells together with polybrene (4 μg/mL). To establish pure population of cells expressing dCas9/TET1, protein selection with blasticidin was performed for 7‐14 days (15 μg/mL).

Jurkat cells with stable dCas9/TET1 expression were transduced with lentivirus particles containing sgRNAs. Nine separate transductions were performed: each sgRNA separately (sg5, sg6, sg7, sg8), mixture of two sgRNAs (sg5,6), mixture of all sgRNAs (sg5,6,7,8), two NT (NT3, NT4) and mixture of two NT (NT3,4). Selection with puromycin was performed for 5‐7 days (2 μg/mL). Cells were harvested for DNA, RNA and protein at three time points—passage 1‐st, 3‐rd and 5‐th post–puromycin selection.

### 2.7 Western Blot

Cells were treated with RIPA lysis buffer (Sigma). Protein concentration was determined using Bicinchoninic Acid Kit (Sigma). Protein samples were mixed with the Laemmli 4X sample buffer (Sigma), denatured and run on the Mini‐PROTEAN Stain‐free gel (Bio‐Rad) with Mini‐PROTEAN® Tetra electrophoresis system (Bio‐Rad). Proteins were semi‐dry transferred onto PVDF membrane (The Mini Trans‐Blot® cell system, Bio‐Rad), blocked and incubated with Cas9 (7A9‐3A3) mouse primary monoclonal antibody (Cell Signaling Technology; Leiden, Netherlands). After incubation with secondary Ab‐HRP (sc‐2005, Santa Cruz Biotechnology), the signal was detected by chemiluminescence with Clarity Western ECL Substrate (Bio‐Rad) using ChemiDoc™ Imaging Systems (Bio‐Rad). Quantitative analysis was performed using ImageLab^TM^ Software. The WB results were normalized using stain‐free technique, by measuring total protein directly on the WB membrane. In this method, trihalo compounds, included in the gel, react with tryptophan residues and after activation by UV light produce fluorescent signal that can be quantified in order to measure the relative amount of sample total protein.

### 2.8 DNA methylation analysis by bisulfite pyrosequencing

The pyrosequencing assay for the analysis of DNA methylation level in *TMEM244* promoter region was designed using the PyroMark Assay Design Software 2.0.1.15 (Qiagen, Hilden, Germany). The assay included the (Forward) 5′‐ AGGATGTTTATTTTGGTATTTA‐GTAGTT‐3′, (Reverse) 5′‐biotin‐labelled‐ AAAATAATAAAAACCCCACTCCT‐3′ and (Sequencing) 5′‐ TTTATTTTGGTATTTAGTAGTTT‐3′ primers. The PCR amplified genomic region (chr6:130,182,353‐130,182,543 GRCh37/hg19) (191 bp) was located upstream of *TMEM244* and overlapped partially the 5’UTR region of the gene (Figure 1). The amplified region overlapped the ENCODE regulatory region as well as several transcription factors binding sites.

DNA methylation level was calculated as the mean methylation measured at three CG dinucleotides at the genomic positions CpG_1 chr6:130,182,513; CpG_2 chr6:130,182,511; and CpG_3 chr6:130,182,486 (GRCh37/hg19) (Figure 1). For the PCR reactions, the PyroMark PCR kit (Qiagen) was used to prepare the following master mix: 12.5 μL PyroMark buffer; 0.5 μL F and R primer each (20 μmol\L); 2.5 μL Coral Load; 8 μL H_2_O; and 1 μL of bisulfite‐converted DNA (in total 25 μL). DNA bisulfite conversion was performed using the EZ DNA Methylation‐Gold™ Kit (Zymo Research, Germany) according to the manufacturer's protocol. The reaction mix was cycled in the following conditions: 95°C for 15 minutes × 1; 94°C for 30 seconds, 59°C for 30 seconds, 72°C for 30 seconds × 45; and 72°C for 10 minutes × 1; 4°C ∞, and the PCR products were visualized on 1.5% agarose gel stained with SimplySafe (EURx) under UV light (BioDoc‐it Imaging System, UVP, USA). The PyroMark Q24 purification station and sequencer were used to obtain single strand DNA and subsequent sequencing as described previously.^16^ Each run included commercially available fully methylated and unmethylated controls (CpGenome™ Human Methylated & Non‐Methylated DNA Standard Set, Sigma‐Aldrich).

## 3 RESULTS

### 3.1 *TMEM44* is expressed in SS and CTCL cell lines with hypomethylation of the promoter region

Our previous study showed that *TMEM244* is expressed in SS patients (P1‐P3 mean ± SD = 681 ± 413E‐6).^11^ In this study, *TMEM244* expression was quantified in different T‐ and B‐cell lymphoma and leukaemia patients, in mononuclear cells of healthy individuals and in four T‐cell lymphoma (TCL) and one TALL cell lines (Table 1). Only trace *TMEM244* expression was detected in healthy individuals (C1‐C5 mean ± SD=19E‐6 ± 13E‐6; BM1 16E‐6) (Table 1), while in majority of T‐cell leukaemia/lymphoma cases and CTCL T‐cell lines the expression of *TMEM244* was present.

*TMEM244* was detected in both SS samples, yet the level was significantly higher for the SS1 patient (SS1 vs SS2 = 8,224E‐6 vs 173E‐6). In MF, that belong to the same group of CTCL lymphomas as SS, the expression of *TMEM244* was detected in blood samples of two patients (mean ± SD MF1‐2PB = 275E‐6 ± 108E‐6), but much lower trace expression was measured in either bone marrow (mean ± SD MF2‐3BM = 52E‐6 ± 4E‐6) or skin biopsy (MF2SB = 8E‐6). Besides CTCLs, one of two non‐cutaneous peripheral T‐cell lymphoma (PTCL) patients and one of three T‐ALL patients showed a meaningful *TMEM244* expression (TCL2: 234E‐6 and TAL2: 165E‐6). The expression was also detected in CTCL cell lines: SeAx (2,536E‐6), HH (363E‐6) and, at a very low level, in Hut78 (50E‐6). HDML2 cell line, derived from a T‐cell Hodgkin lymphoma, had a relatively high expression of *TMEM244* (1,482E‐6), while no expression was detected in Jurkat cells.

All but one B‐cell leukaemia samples (10/11) showed only a trace *TMEM244* expression (mean ± SD=15.3E‐6 ± 18.3E‐6), suggesting that this gene may be exclusive for T‐cell malignancies. In one CLL sample, otherwise not different, a meaningful expression was detected (CLL5: 433E‐6).

In order to determine whether *TMEM244* expression is regulated by DNA methylation, three CpG dinucleotides in the *TMEM244* promoter region were analysed by bisulfite pyrosequencing. The results showed that in cells without or with trace *TMEM244* expression the CpG sites were highly methylated, including healthy individuals (C1‐C5 mean ± SD=86.68% ± 1.58; BM1 = 91.44%), Jurkat cell line (85.5%) and most B‐cell leukaemias (mean ± SD = 88.3% ± 6.8). On the contrary, in cells expressing *TMEM244* at a significant level the promoter was hypomethylated. In homogeneous cell lines with high *TMEM244* expression, like SeAx, HDLM2 and HH, the promoter was completely demethylated (1.9%, 1.8%, 3.2% respectively). Similar effect was observed for SS1. This sample consisted of a homogenous population of malignant CD4 + lymphocytes with the highest expression of *TMEM244* detected in our study and complete demethylation of its promoter region (4.3%). In SS2 and Hut78, lower *TMEM244* expression was observed, and methylation analysis showed that it is a result of partial demethylation of *TMEM244* promoter (33.4% and 56% respectively). Three samples from a previous study (P1‐P3) consisted of a heterogeneous population of mononuclear cells. Therefore, despite 33.5%‐67.9% methylation, most likely derived from the admixture of non‐malignant cells, the samples showed high *TMEM244* expression. In 2/3 MF patients, partial demethylation of *TMEM244* promoter in the blood samples (59% and 70%) was accompanied by a moderate *TMEM244* expression (198E‐6 and 351E‐6). In one PTCL and one T‐ALL samples with a weak *TMEM244* expression, the mean methylation level was only slightly decreased compared with controls (73% and 80% respectively).

**FIGURE 2:**
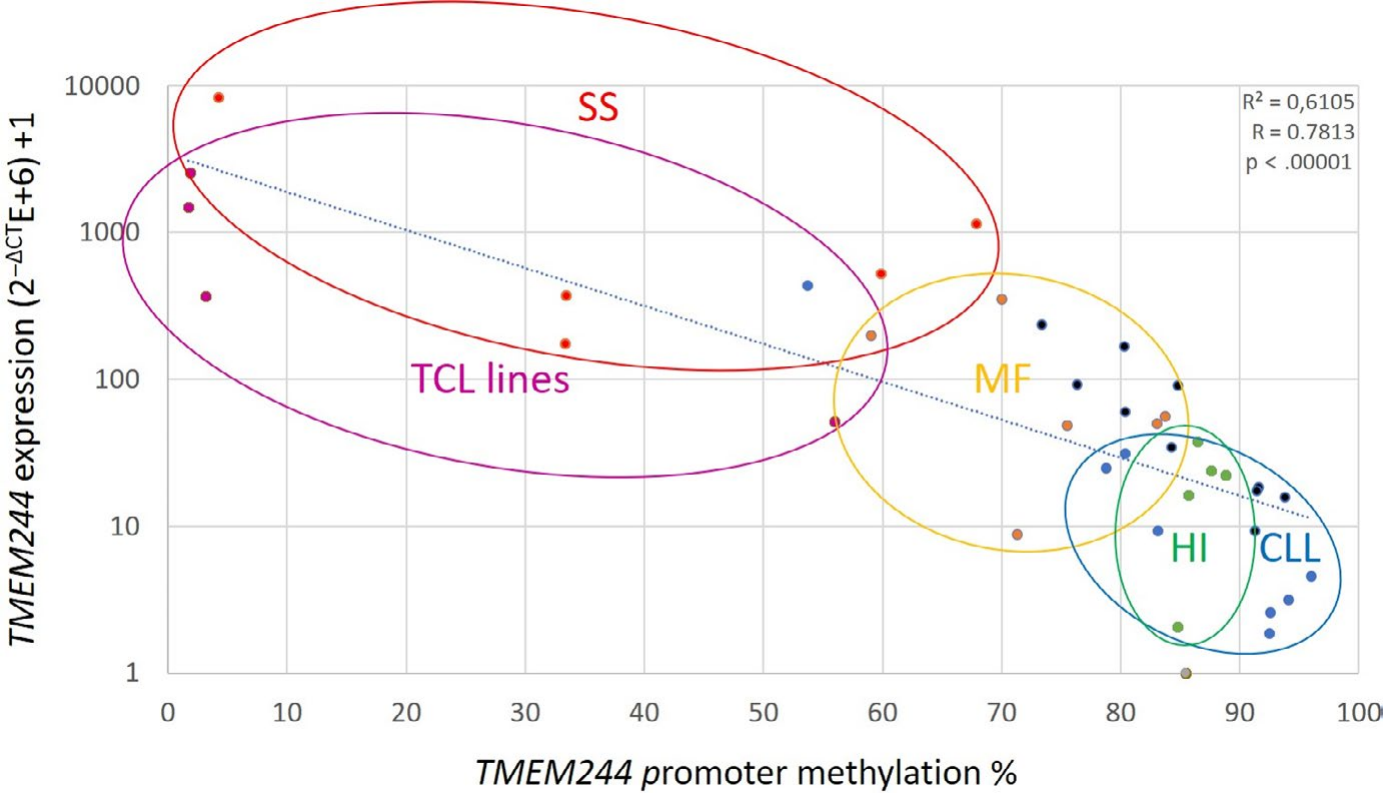
Correlation between promoter DNA methylation and *TMEM244* expression in lymphoid malignancies and in T‐cell lines (SS: Sézary syndrome—red, MF: Mycosis fungoides—orange, CLL: chronic lymphoblastic leukaemia—blue, HI: healthy individuals—green, T‐cell lymphoma cell lines—violet)

Among the 39 samples collected from patients, healthy donors and cell lines, a meaningful *TMEM244* expression was observed in 13 samples: five SS, two peripheral blood of MF, one CLL, one T‐ALL, one T‐cell lymphoma, and three T‐cell lines. In those samples, the mean promoter methylation level was 44.11%±30.4 and the mean *TMEM244* expression was 1,246E‐6 ± 2,206E‐6. In the samples with trace or no *TMEM244* expression (27E‐6 ± 26E‐6), the mean methylation level was markedly higher (85% ± 8.5). Based on the obtained results, a cut‐off value for promoter hypomethylation was set at 70% and for *TMEM244* expression at 100E‐6. Using these cut‐offs, 11/12 samples with promoter hypomethylation expressed *TMEM244* and 25/27 samples with methylated promoter did not express *TMEM244* (*P* < 0.000001 in Fisher exact test; Table 1). Pearson correlation coefficient test showed a strong negative correlation between *TMEM244* expression and the square of its promoter methylation (Figure 2; *R* = −0.7813; *P* < .00001).

### 3.2 In vitro demethylation of *TMEM244* promoter activates *TMEM244* expression

**FIGURE 3:**
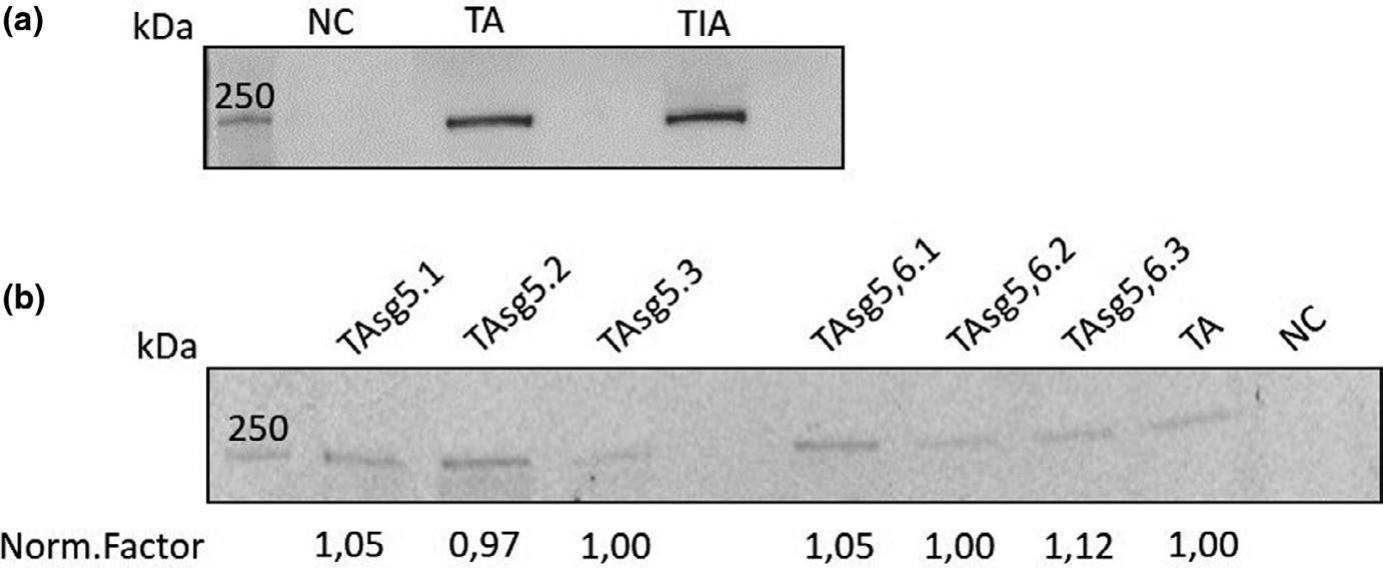
Western blot analysis of dCas9‐TET1 expression. (a) Confirmation of dCas9‐TET1 fusion protein expression in transduced Jurkat cells, (b) Quantitative analysis of dCas9‐TET1 expression over time. NC: negative control (JWT); TA: TET1 active domain; TIA: TET1 inactive domain.; TAsg: TET1 active domain with single guided RNA; .1, .2, .3: time points

In order to prove the mechanism of *TMEM244* transcriptional activation by promoter demethylation, the CRISPR‐dCas9‐TET1 system was used for directed demethylation. In the first step, Jurkat cell line was transduced with the dCas9‐Tet1 expressing vector. After selection, the expression of this fusion was confirmed on protein level using Western blot and anti‐dCas9 antibody (Figure 3A). Secondly, cells were transduced with vectors expressing sgRNAs. sgRNAs were used separately, or in combination of 2 or 4, as described in the Section 2. The GFP marker was used to confirm sgRNAs expression. Cells were harvested for analysis at three different time points that were stated as passage 1, 3, and 5 post–antibiotic selection. At each time point, the protein level was evaluated and the quantitative analysis using stain‐free technology showed that the expression of dCas9‐TET1 fusion protein was stable over time (Figure 3B). At each time point, the expression of *TMEM244* mRNA was checked, as well as methylation level of three CpG dinucleotides in the promoter region. Overall, analysis of *TMEM244* expression and promoter methylation level of all samples, in all time points, showed that the specific sgRNAs with the dCas9‐TET1 complex decreased DNA methylation level in the studied region and activated the expression of *TMEM244*. In contrast, inactive TET1 did not affect methylation of chosen CpG dinucleotides (Supplementary).

Four sgRNAs, targeting different sites in the promoter region, were used. Transduction with sg5 and sg6 resulted in a significant reduction of methylation level by 30% and 25% respectively. Acting together the decrease was only by 10%. No effect was detected for two other sgRNAs, sg7 and sg8, and also for two NTs. Decreased DNA methylation was accompanied by activated *TMEM244* expression in Jurkat cells. The expression was low; however in wild‐type Jurkat (JWT), *TMEM244* expression was completely absent.

**FIGURE 4:**
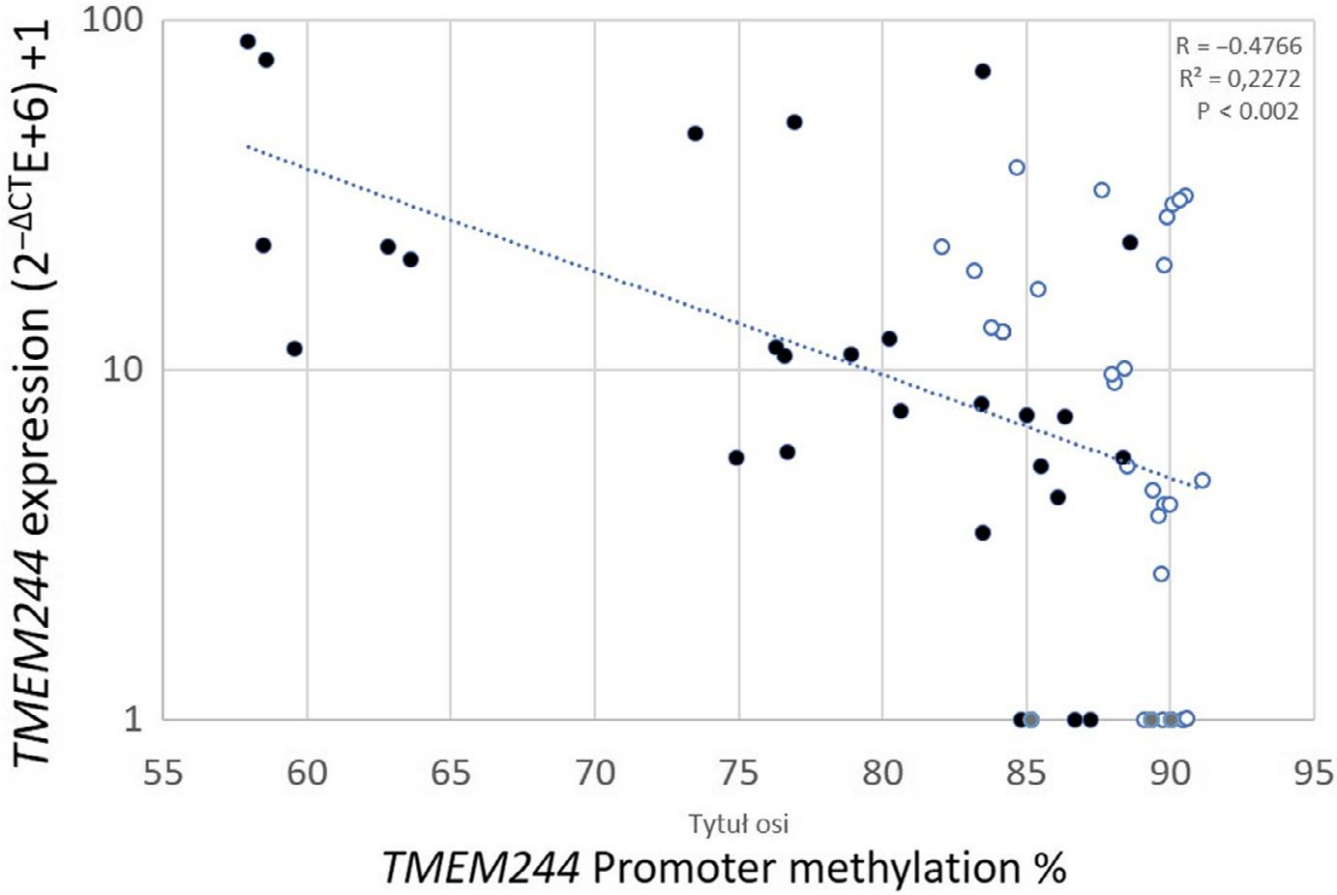
Correlation between methylation and *TMEM244* expression. Black dots: TET1 active; empty dots: TET1 inactive; grey dots: no sg

To check the correlation between *TMEM244* expression and promoter methylation, we performed the Pearson correlation coefficient test (Figure 4) for all dCas9‐TET1 samples. The analysis showed that the level of *TMEM244* expression is negatively correlated with the square of the methylation level in the promoter region (*R* = −0.4766), and this correlation is highly significant (*P* < 0.0002) (Figure 4).

## 4 DISCUSSION

In this study, we showed that methylation is a key regulatory mechanism of *TMEM244* expression. Samples with *TMEM244* expression, among them mostly SS and a few other T‐cell leukaemia/lymphoma cases, had promoter region hypomethylated, while in all samples not expressing the gene, the promoter was methylated. The negative correlation between *TMEM244* expression and promoter methylation was confirmed, and the mechanism was verified using CRISPR‐dCas9‐TET1 system for directed demethylation of the specific sites in the promoter region. This approach has not been used in CTCL studies so far. The only methylation modification was performed using 5‐aza‐2'deoxycitidine, a pan‐demethylating compound. Upon 5‐aza treatment, a down‐regulated expression of two tumour suppressors, *THBS4* and *PTPRG* were restored,^9^ as well as a potential epigenetic diagnostic marker CMTM2^8^ and miR200c involved in activation of Notch pathways in CTCL.^17^ The advantage of using CRISPR‐dCas9‐TET1 approach is its specificity to the region of interest, without affecting global methylation patterns. Therefore, the observed expression activation can be directly correlated with the *TMEM244* promoter methylation changes introduced by TET1 protein. In our study, we managed to ‘switch on’ the expression in Jurkat cell line with no basal *TMEM244* expression by demethylation using TET1 fused to dCas9 and guided by sgRNAs. sgRNAs design is the crucial step for CRISPR‐Cas9 technology. In our case, the best results were obtained for sgRNAs that were localized closely to CpGs sites, no further than 100 bp. Two sgRNAs situated > 100 bp form CpGs did not have the expected effect. Contrary to the reports showing enhanced effect of combining two or more sgRNAs, in our hands two sgRNAs were less efficient than individual sgRNAs.

Genome‐wide methylation analysis in CTCLs revealed that more CpG sites were hypomethylated than hypermethylated.^8^, ^18^ Hypomethylation leads to chromosomal instability and is often observed in cancer genomes. However, there are only two reports that actually describe a hypomethylation of specific genes in CTCLs. Wong et al described hypomethylation‐mediated overexpression of *PLS3*, *GATA3* and *TWIST3*.^18^ *GATA3* overexpression in CTCL was confirmed by Kamijo et al^19^ The study showed that hypomethylation‐mediated *GATA6* overexpression promotes tumour progression via overexpression of *CD137L* that together with *CD137* activates pathways leading to cell proliferation, tumour survival, growth and migration.

More studies were published on hypermethylated genes in CTCL, as they are often tumour suppressor genes involved in DNA repair, cell cycle, proliferation and apoptotic pathways. Hypermethylation in the promoter region, followed by decreased expression level, was detected for several tumour suppressors, including *CDKN2B* (p15), *CDKN2A* (p16) and *MGMT*,^20^ *BCL7A*, *PTPRG* and *TP73* (p73)^9^ and *RUNX3*/p46.^21^ Promoter methylation not always resulted in gene silencing, and overexpression of *IL‐15* in *CTCL* was actually associated with hypermethylation of the promoter, preventing binding of ZEB1 transcription repressor.^22^

Little is known about the *TMEM244* gene itself. It belongs to a family of transmembrane proteins (TMEMs) that are components of various membranes (cell membranes, mitochondrial, ER, lysosomal, Golgi membranes), present in different cells and fulfil important physiological functions. Many TMEMs are differentially expressed in different cancers.^23^ So far, the role of *TMEM244* is unknown and no studies have been conducted in order to unravel its function. Although many RNAseq analysis has been performed for CTCLs samples,^4^, ^24^, ^25^ only our team paid attention to that gene, probably due to its relatively low expression.

Our current results show that the expression of *TMEM244* gene is associated with T‐cell lymphomas, especially with Sézary syndrome, and is a result of specific hypomethylation of its promoter. Since the expression of *TMEM244* and the hypomethylation of its promoter are specific to T‐cell lymphoma, with the highest expression in SS, they could be used as a diagnostic marker in this type of CTCL.

## CONFLICTS OF INTEREST

The authors confirm that there are no conflicts of interest.

## AUTHORS' CONTRIBUTION

KI planned the experiments, drafted the manuscript and together with KR carried out the main part of the research, including expression analysis and in vitro experiments. KN isolated RNA and DNA; M.Ż. and I.Z‐S. produced the lentiviral vectors; MP performed the Western blot; A. D‐K. contributed to the study design, interpretation of the results and preparation of the final version of the manuscript; JP carried out the methylation studies; MJ and K. O‐H. provided patient samples along with the clinical laboratory data and clinical consultation; MG designed the methylation analysis, evaluated the results and contributed to the final version of manuscript; and GKP conceived the study, was in charge of overall direction and planning and prepared the final version of the manuscript. All authors provided critical feedback and helped shape the final version of the manuscript.

## Data Availability

The data that support the findings of this study are available in the supplementary material of this article.

## References

[1] Willemze R , Jaffe ES , Burg G , et al. WHO‐EORTC classification for cutaneous lymphomas. Blood. 2005;105:3768–3785. 15692063 10.1182/blood-2004-09-3502

[2] Campbell JJ , Clark RA , Watanabe R , Kupper TS . Sezary syndrome and mycosis fungoides arise from distinct T‐cell subsets: a biologic rationale for their distinct clinical behaviors. Blood. 2010;116:767–771. 20484084 10.1182/blood-2009-11-251926 PMC2918332

[3] Bastidas Torres AN , Najidh S , Tensen CP , Vermeer MH . Molecular advances in cutaneous T‐cell lymphoma. Semin Cutan Med Surg. 2018;37:81–86. 29719024 10.12788/j.sder.2018.007

[4] Wang L , Ni X , Covington KR , et al. Genomic profiling of Sezary syndrome identifies alterations of key T cell signaling and differentiation genes. Nat Genet. 2015;47:1426–1434. 26551670 10.1038/ng.3444 PMC4829974

[5] Choi J , Goh G , Walradt T , et al. Genomic landscape of cutaneous T cell lymphoma. Nat Genet. 2015;47:1011–1019. 26192916 10.1038/ng.3356 PMC4552614

[6] Kiel MJ , Sahasrabuddhe AA , Rolland DCM , et al. Genomic analyses reveal recurrent mutations in epigenetic modifiers and the JAK‐STAT pathway in Sezary syndrome. Nat Commun. 2015;6:8470. 26415585 10.1038/ncomms9470 PMC4598843

[7] da Silva Almeida AC , Abate F , Khiabanian H , et al. The mutational landscape of cutaneous T cell lymphoma and Sezary syndrome. Nat Genet. 2015;47:1465–1470. 26551667 10.1038/ng.3442 PMC4878831

[8] van Doorn R , Slieker RC , Boonk SE , et al. Epigenomic analysis of sezary syndrome defines patterns of aberrant DNA methylation and identifies diagnostic markers. J Invest Dermatol. 2016;136:1876–1884. 27113428 10.1016/j.jid.2016.03.042

[9] van Doorn R , Zoutman WH , Dijkman R , et al. Epigenetic profiling of cutaneous T‐cell lymphoma: promoter hypermethylation of multiple tumor suppressor genes including BCL7a, PTPRG, and p73. J Clin Oncol. 2005;23:3886–3896. 15897551 10.1200/JCO.2005.11.353

[10] Shen H , Laird PW . Interplay between the cancer genome and epigenome. Cell. 2013;153:38–55. 23540689 10.1016/j.cell.2013.03.008 PMC3648790

[11] Izykowska K , Przybylski GK , Gand C , et al. Genetic rearrangements result in altered gene expression and novel fusion transcripts in Sezary syndrome. Oncotarget. 2017;8:39627–39639. 28489605 10.18632/oncotarget.17383 PMC5503638

[12] Salimi M , Subramaniam S , Selvakumar T , et al. Enhanced isolation of lymphoid cells from human skin. Clin Exp Dermatol. 2016;41:552–556. 26805629 10.1111/ced.12802 PMC4981906

[13] Kaltoft K , Bisballe S , Rasmussen HF , Thestrup‐Pedersen K , Thomsen K , Sterry W . A continuous T‐cell line from a patient with Sezary syndrome. Arch Dermatol Res. 1987;279:293–298. 3498444 10.1007/BF00431220

[14] Liu XS , Wu H , Ji X , et al. Editing DNA methylation in the mammalian genome. Cell. 2016;167:233–247. 27662091 10.1016/j.cell.2016.08.056 PMC5062609

[15] Chen B , Gilbert L , Cimini B , et al. Dynamic imaging of genomic loci in living human cells by an optimized CRISPR/Cas system. Cell. 2013;155:1479–1491. 24360272 10.1016/j.cell.2013.12.001 PMC3918502

[16] Szaumkessel M , Richter J , Giefing M , et al. Pyrosequencing‐based DNA methylation profiling of Fanconi anemia/BRCA pathway genes in laryngeal squamous cell carcinoma. Int J Oncol. 2011;39:505–514. 21567085 10.3892/ijo.2011.1039

[17] Gallardo F , Sandoval J , Díaz‐Lagares A , et al. Notch1 pathway activation results from the epigenetic abrogation of notch‐related MicroRNAs in mycosis fungoides. J Invest Dermatol. 2015;135:3144–3152. 26302069 10.1038/jid.2015.328

[18] Wong HK , Gibson H , Hake T , et al. Promoter‐specific hypomethylation is associated with overexpression of PLS3, GATA6, and TWIST1 in the sezary syndrome. J Invest Dermatol. 2015;135:2084–2092. 25806852 10.1038/jid.2015.116

[19] Kamijo H , Miyagaki T , Shishido‐Takahashi N , et al. Aberrant CD137 ligand expression induced by GATA6 overexpression promotes tumor progression in cutaneous T‐cell lymphoma. Blood. 2018;132:1922–1935. 30194255 10.1182/blood-2018-04-845834

[20] Gallardo F , Esteller M , Pujol RM , Costa C , Estrach T , Servitje O . Methylation status of the p15, p16 and MGMT promoter genes in primary cutaneous T‐cell lymphomas. Haematologica. 2004;89:1401–1403. 15531468

[21] Haider A , Steininger A , Ullmann R , et al. Inactivation of RUNX3/p46 promotes cutaneous T‐cell lymphoma. J Invest Dermatol. 2016;136:2287–2296. 27377697 10.1016/j.jid.2016.05.126

[22] Mishra A , La Perle K , Kwiatkowski S , et al. Mechanism, consequences, and therapeutic targeting of abnormal IL15 signaling in cutaneous T‐cell lymphoma. Cancer Discov. 2016;6:986–1005. 27422033 10.1158/2159-8290.CD-15-1297 PMC5388135

[23] Schmit K , Michiels C . TMEM proteins in cancer: a review. Front Pharmacol. 2018;9:1345. 30574087 10.3389/fphar.2018.01345 PMC6291505

[24] Lee CS , Ungewickell A , Bhaduri A , et al. Transcriptome sequencing in Sezary syndrome identifies Sezary cell and mycosis fungoides‐associated lncRNAs and novel transcripts. Blood. 2012;120:3288–3297. 22936659 10.1182/blood-2012-04-423061 PMC3476540

[25] Prasad A , Rabionet R , Espinet B , et al. Identification of gene mutations and fusion genes in patients with sezary syndrome. J Invest Dermatol. 2016;136:1490–1499. 27039262 10.1016/j.jid.2016.03.024

